# Development and Implementation of a Preventive Intervention for Youth with Concerns About Their Sexual Thoughts and Behaviors: A Practitioner Narrative

**DOI:** 10.1007/s10935-023-00758-8

**Published:** 2023-12-01

**Authors:** Melissa Bright, Brittany Gordon, Csenge Bodi, Diana Ortega, Jennifer Coleman

**Affiliations:** 1 Center for Violence Prevention Research, Gainesville, FL USA; 2 Stop it Now!, Northampton, MA USA

**Keywords:** Youth sexual behavior, Primary and secondary prevention, Helpline, Child sexual abuse, Sexual harm

## Abstract

This practitioner narrative describes the development of an innovative, primary and secondary prevention resource to provide confidential resources to youth with questions about potentially problematic sexual interests and behaviors. WhatsOK is a website and free confidential helpline for youth who are potentially at risk to sexually harm or have harmed someone in the past. By encouraging self-efficacy, helpline counselors respond to these inquires in order to prevent harmful events or lessen the impact. This practitioner narrative begins with an explanation of the planning process, then describes the implementation, piloting and refining the resource, and, finally, explains how evaluation was incorporated. The development of the WhatsOK helpline services was conducted with the goal of creating an evidence-informed resource for youth with concerns about sexual thoughts and behaviors.

In 2021, Stop it Now! (Now!), a United States-based, non-profit child sexual abuse (CSA) primary perpetration prevention organization, launched an online resource and confidential helpline—WhatsOK—for youth and young adults with concerns about potentially problematic sexual interests and behaviors. Now!’s original helpline was developed to provide resources and divert *adults* from committing sexual offenses towards children. However, in 2019, 11% of inquiries were youth asking for help with their sexual interests and behaviors towards younger children. Consistent with recent literature, youth contribute significantly as perpetrators of CSA. Some estimate that 70% or more of sex offenses are committed by other youth (Gewirtz-Meydan & Finkelhor, 2020), with the average age of first-time sexual perpetration being approximately 14–16 years (Ybarra & Mitchell, 2013). Moreover, nearly 1 in 10 youth report some type of self-involved sexual perpetration (Finkelhor et al., 2009; Ybarra & Mitchell, 2013). Despite the significant need for support and information, resources for youth and young adults with potentially problematic sexual interests and behaviors are limited.

WhatsOK aims to (1) increase knowledge of CSA and CSA prevention, (2) change perceptions of availability and utility of support resources, (3) encourage self-efficacy in preventing CSA, and (4) increase the use of protective and interventional behaviors when risk factors are identified. Among the reasons why youth may sexually abuse another child or engage in sexual misconduct harmful to others are the following: feeling sexual attraction for children, trauma reactive behaviors, lack of healthy sexuality knowledge, developmental delays, emotional intelligence differences including spectrum diagnoses, drug/alcohol use, or other psychiatric disabilities (Finkelhor et al., 2009). Increased access to online pornography is also now often part of a youth’s introduction to sexuality (Horvath et al., 2013). Ongoing viewing of pornography can impact a young person’s image of sexual behaviors, influencing perspective on what is safe, healthy, and consensual (Huntington et al., 2022). During consideration of the development of a youth-targeted resource, most inquiries to the Now! helpline primarily focused on: (1) worries about online addiction to child pornography, (2) fears that reaching out for help will label them for life, and (3) depression related to a sense of hopelessness regarding a “normal” life.

An example email from a youth at risk to abuse:

“I have noticed that I have an attraction to prepubescent girls (and) feel that their safety around me were quickly dwindling, and it had become exponentially harder for me to resist. I have told my mother, but she doesn’t know what [to] do and [was] as depressed about it as I am. I’m not entirely sure what to do, but I do know that there is no cure, so what I’m asking for were ways to prevent the provocation, if it were possible. It would really help if I can receive fast help.”

This narrative describes the development of an innovative, primary and secondary prevention resource to provide confidential resources to youth with questions about potentially problematic sexual interest and behaviors. We begin with an explanation of the development process, then describe the implementation, piloting and refining the resource, and finally how evaluation was incorporated from the beginning.

## Development of a Resource for Youth

The Now! team created a small project team including people in the following areas of expertise: child sexual abuse prevention, research, evaluation, social media, and marketing. Funding to support development and pilot testing was obtained through competitive application with the World Childhood Foundation.

**Fig. 1:**
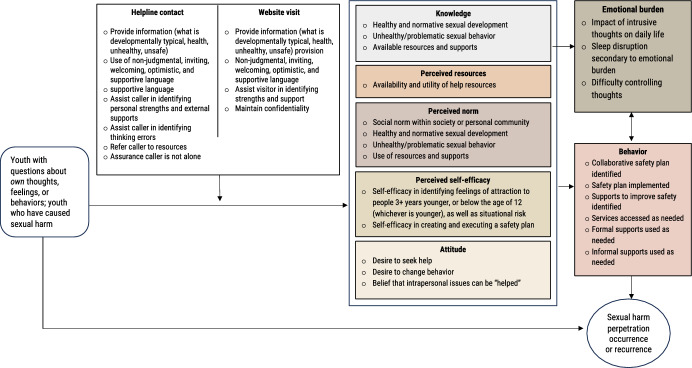
Logic model for WhatsOK helpline and website services for youth with potentially problematic sexual behaviors

The logic model behind WhatsOK is based on the Theory of Planned Behavior (Ajzen, 1991) which posits that behavior is determined by a person’s knowledge of the behavior and how to complete it, their attitudes toward that behavior, their perceived resources and self-efficacy to complete the behavior, and their perceived social norms around completing the behavior. In the context of this project, the target behaviors are seeking formal therapeutic supports and not engaging or re-engaging in sexual harm (Fig. 1). The proposed theory of WhatsOK influencing these behaviors is that youth with potentially problematic sexual behaviors who receive information in a supportive, non-judgmental setting will have improved knowledge of healthy and unhealthy sexual behavior, will be able to identify resources, will understand social norms around sexual behavior, will have greater self-efficacy in finding resources and changing behavior, and will want to seek further help.

### Website and Helpline

The WhatsOK.org site includes 8 pages: a homepage, a contact page, a page explaining how the helpline works, a 5-part FAQs page, a blog, a reference page of additional resources, an about page, and a privacy policy page. Each webpage was developed custom for the youth audience, including age-appropriate guidance for pre-adolescents, adolescents, and young adults, while incorporating Now!’s signature tone of hopefulness, accountability and support. Website content was reviewed by our internal team of experts as well as several external experts.

Individuals can contact the WhatsOK helpline via email, phone, text, online chat, or postal mail and speak confidentially to a helpline counselor. The Now! team trained three internal employees as helpline counselors to respond to youth-specific inquiries. All counselors had backgrounds in healthy sexuality, trauma, and/or counseling. Training topics included motivational interviewing with youth, strategizing resources and safety planning with youth, and deterrence planning for CSAM viewing. Content relevant research and resources were reviewed and discussed, such as pornography viewing behaviors, sexting, and varied types of anime. Ongoing supervision and consultation were provided.

### Experts and Youth Advisors

The Now! team also sought input from experts in the fields of child sexual abuse and sexual harm. Now! received input from the National Center on the Sexual Behavior of Youth (NCBSY), a clinician specializing in the treatment of sexually harmful behaviors, professionals with a national youth hotline, an international organization that helps web-based companies prevent child sexual abuse, a noted journalist with connections to youth who are identifying as minor attracted persons (MAP), academic researchers with expertise in preventing harmful youth sexual behaviors, a marketing company with experience developing campaigns around healthy relationships, and a university-based, student-led organization that supports students who have experienced sexual harm.

Including the youth voice was critical to development of WhatsOK. The Now! team created a Youth Advisory Council consisting of ten youth aged 14–21 years. Some youth council members had a history of sexual behavior problems, including a young man incarcerated for viewing child sexual abuse material (CSAM), and youth who were previously part of a treatment group for sexual problem behaviors were also recruited. Other council members were part of an advocacy youth group at a local high school group, and some others were recruited through professional networks. The Youth Advisory Council was asked for input on, among other topics, website content and resources, media campaign messages, and engaging with youth through text and chat.

### Youth-Focused Social Media Advertisements

The Now! team created 4 youth-focused advertisements for social media outreach (Bright et al., 2023). Advertisements were designed by a marketing expert based on successful strategies used by other organizations that focused on similar topics. That is, advertisements were brightly colored, included short questions, and primarily used graphics instead of images of people.

### Evaluation

The Now! team included a researcher since initiating resource development to ensure the creation of an evidence-informed service. The researcher conducted formative analyses on the process of developing WhatsOK including tracking results from alpha, beta, feasibility, and usability testing. The project team met monthly to discuss patterns in advertisement performance, website traffic, number and nature of contacts, and updates on dissemination. Issues with any of the components were discussed and resolved during these meetings. The data management system was also adapted, and the project team continues to evaluate and refine data management processes.

## Launch

The helpline and social media campaign launched in October of 2021. The Now! team distributed announcements of the youth-focused helpline services to thousands of individuals and agencies globally, using their contact list of individuals and national listservs, coalitions, and state and federal agencies. As of April 2023, approximately 1.5 years since the launch, the Now! team reached 1,959,021 youth/young adults through social media and achieved 4,656,736 impressions (i.e., views of an ad), 47,481 engagements, and 2,179 shares. WhatsOK.org was accessed by 62,316 users who collectively viewed pages 113,174 times across 70,271 sessions. WhatsOK helpline counselors responded to 558 inquiries of which approximately 54% were individuals who had or were at risk to cause sexual harm.

### Challenges and Lessons Learned

The development of the WhatsOK helpline services presented some challenges. One challenge was determining what resources are available to youth that do not require parental consent. For example, how do helpline counselors recommend therapy if the youth does not want their parent/guardian to know but the insurance is through the parent/guardian? In addition, most mental health resources require parental consent for youth under 18. Interpreting and understanding laws about fictional sexual content, including hentai, lolicon, and shotacon were also challenges. It is important for Now! staff to stay current in their knowledge of sexualized fictional content—both the laws around this content and the impact on youth and young adults. The laws around this content are constantly changing and evolving and can vary by state or internationally. A third significant challenge was developing content for the wide age range of the target audience (14–21 years). For example, certain sexual behavior may be developmentally appropriate, healthy, and legal for a 14-year-old but developmentally inappropriate or illegal for a 20-year-old.

We anticipate new challenges will arise as this new service is disseminated broadly and usage increases. Most challenging will be the need to appropriately match the number of helpline staff to the rate of inquiry growth. We will need to closely monitor the number of missed contacts to determine the best time to increase helpline hours and, if necessary, hire more helpline counselors. Relatedly, finding funding for a free service will always be a challenge. We will need to explore private and government funding sources with the goal of obtaining significant and consistent funding.

The launch of WhatsOK provided many opportunities for further growth. First and foremost, youth who contact the helpline are articulate, thoughtful, inquisitive, and responsible. Youth who contacted the helpline communicated their needs and concerns in thoughtful ways. Today, youth are turning to the internet to find answers specific to their sexual thoughts and behaviors, whether it be through social media or other online discussion platforms, such as Reddit. Youth who contacted the WhatsOK helpline were considering the risks of their behaviors and seeking information to help them in their decision making. It was apparent that these youth were also aware of their potentially problematic behaviors, interests, and feelings. It is important to recognize that users of WhatsOK may not represent all youth with potentially problematic sexual thoughts, feelings, or behaviors. Instead, they represent youth who are a) aware of their potentially problematic sexual thoughts, feelings, or behaviors, are b) aware of the service, and are c) motivated to seek help through the website helpline. Youth with potentially problematic sexual behaviors who are not aware of the problematic nature of their behaviors, are not aware of support services, or are not interested in receiving services may have categorically different needs from youth who used the WhatsOK service. Identifying and addressing these differences are ongoing goals for Now! Youth who contacted the helpline also inquired about approaching someone they felt they may have harmed. WhatsOK offers youth the opportunity take accountability and navigate the necessary steps to remedy a situation for which they feel responsible.

The Now! team has developed more understanding of both the experiences and the challenges facing youth specific to their digital media and internet use. This includes recognizing that information available for youth regarding their viewing behaviors, especially when it includes things like fictional sexual content, are limited and, sometimes, difficult to understand. For example, while youth want to know if their viewing behaviors regarding this content is legal, this remains a gray area in the U.S. Youth’s exposure to sexualized online content that does not identify as CSAM, has an impact on the sexual interests of youth. This includes fictional sexual content, such as hentai, lolicon, shotacon, and other forms of anime. Many youths who contacted the helpline expressed that they had seen this content, and felt the urge to look at it further, even when it might not align with their sexual attractions. They also expressed that looking at this content was affecting their relationships, or their thoughts around this content felt unmanageable.

It’s also important to note that many youths felt concerned about what is legal, especially regarding age of consent. Questions such as “Is it okay to date someone who is X years younger than me?” were common. Youth also wanted to understand the impact of their experiences on their own behaviors. They understood that their own experiences of abuse may impact how they experience relationships, their bodies and sexuality. However, they expressed uncertainty when considering strategies to address short-term and long-term impacts.

## Conclusion

WhatsOK is the first, and only US-based helpline to offer practical deterrent services to at-risk youth, offering new services for this vital population, confidentially gaining new insights about and from them, and sharing new findings and resources with the abuse prevention field.

The WhatsOK helpline aims to reduce the impact of problematic sexual behaviors by allowing youth to ask questions and directing them to appropriate resources. Having this opportunity to talk to helpline counselors who encourage protective behaviors may help prevent potentially harmful sexual behaviors. From a primary and secondary prevention standpoint, the WhatsOK helpline assists youth who are at risk to harm, as well as those you may have already harmed someone.
